# Association between daily screen time and risk of stroke among middle-aged and elderly people: research based on China health and nutrition survey

**DOI:** 10.3389/fspor.2023.1307930

**Published:** 2023-12-13

**Authors:** Yaxin Ren, Kejuan Sun, Yueqing Rong, Shiming Song, Yijing Zhai, Junjie Miao, Hongmei Shi, Hongmei Xue, Zengning Li

**Affiliations:** ^1^ Department of Clinical Nutrition, The First Hospital of Hebei Medical University, Shijiazhuang, Hebei, China; ^2^ Hebei Key Laboratory of Nutrition and Health, Shijiazhuang, Hebei, China; ^3^ Department of Clinical Nursing, The First Hospital of Hebei Medical University, Shijiazhuang, Hebei, China; ^4^ School of Public Health, Hebei Medical University, Shijiazhuang, Hebei, China

**Keywords:** screen time, stroke, sedentary behavior, energy intake, physical activity

## Abstract

**Background:**

We aimed to explore the independent associations between screen time and the risk of stroke among Chinese adults based on the China Health and Nutrition Survey (CHNS).

**Methods:**

Data on Chinese adults aged older than 40 years from the CHNS in during 2004–2009 were selected. A total of 4,587 individuals were included in 2009, including screen time and the risk of stroke. Simultaneously, we traced the previous screen time to 2004 for those with outcome measures in 2009 (*n* = 2,100). Basic information, lifestyle, and screen behavior were obtained through face-to-face interviews and self-completed questionnaires. Anthropometric data collected included blood pressure, body weight, height, hip circumference, and waist circumference. Fasting blood was obtained for measurements of lipid and glucose levels. Cross-sectional analysis and cohort analysis were both performed using multivariate logistic regression.

**Results:**

Of all participants, 3,004 (65.49%) participants spent more than 2 h per day on screen time. Taking the men who spent less than 2 h on screen per day as reference, the crude odds ratio (OR) of the high risk of stroke was 1.53 [95% confidence interval (CI), 1.20–1.95] for the men who spent 2–3 h per day on screen and 2.37 (95% CI, 1.78–3.16) for the men who spent more than 3 h per day on screen. This difference remained significant after adjusting for confounding factors. No association was observed among women. However, in the cohort analysis with screen time in 2006 as the independent variable, the association between screen time and stroke risk was found both in men [OR, 1.83 (95% CI, 1.19–2.82)] and women [OR, 1.48 (95% CI, 1.10–1.99)]).

**Conclusion:**

We found that the high screen time was associated with an increased stroke risk, which was pronounced in men, warranting a universal need to limit screen time in order to improve health.

## 1. Introduction

Stroke is the second leading cause of disability and death worldwide, with more than 13 million new cases each year (1, 2). From 1990 to 2016, the absolute number of people who died or became disabled from stroke nearly tripled (3). Current studies have found a variety of risk factors for stroke, like hypertension (4), diabetes (5), hyperlipidemia (6), unhealthy lifestyle (7), and family history of stroke (8). Contemporary people spend nearly two-thirds of their leisure time on screen-based sedentary activities (9) and lifestyle were modifiable (10, 11).

Studies have found associations between sedentary behavior or screen time and stroke risk factors (12–14). In the 2003–2004 National Health and Nutrition Examination Survey (NHANES), older adults were the number one sedentary group (13). Among adults from 10 European countries, those who watched 1 h or more of TV per day had an increased risk of total cardiovascular disease (CVD) (15). Stamatakis et al. (16) found the risk of CVD being 2.25 times higher for people with more than 4 h of screen time per day than those with less than 2 h. In an Australian study, participants who had their first stroke managed to watch significantly more TV than those who did not have a stroke (17). But after adjusting for age and sex, this association disappeared (17). These studies focused on the relationship between total cardiovascular disease and screen time. There are very few studies on the relationship between screen time and stroke, a specific cardiovascular disease.

In China, people now spend much more time on the screen every day. A study focused on Chinese adults (18) found that more than 40% of people over the age of 45 watched more than 2 h of TV a day. There are some studies (19–21) on sedentary time/screen time and CVD/stroke in China and the results show that they are closely related. However, these studies mainly focused on the relationship between overall cardiovascular diseases and screen time, or the connection between stroke and prolonged sitting. There have been no studies specifically targeting the relationship between stroke and screen time. This means that we still know very little about the association between stroke and screen time. Furthermore, these studies are regional and the study population is limited in scope. Our study focused on the national population, making it more representative and revealing more fully the relationship between stroke and screening time. The proportion of screen time is likely to increase in the future, so research on stroke and screen time is essential.

Given the limited research on the relationship between screen time and stroke in adults, we studied the distribution and the impact of screen time on stroke in the Chinese population using data from the China Health and Nutrition Survey (CHNS).

## 2. Methods

### 2.1. Data source and study participants

**Figure 1:**
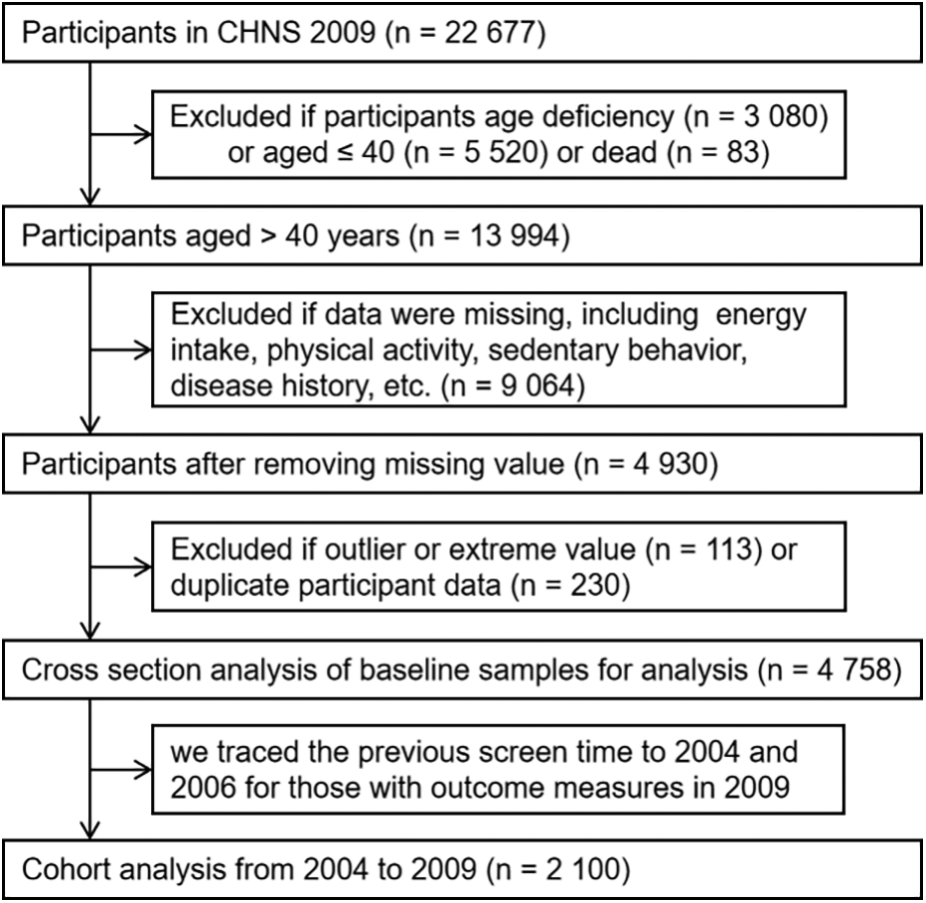
Flow chart of sample selection from the CHNS.

The data were obtained from the China Health and Nutrition Survey, a national surveillance database by consistently collecting a dataset using a standard protocol from the nationally standardized electronic medical records across hospitals in mainland China (22). Our study's population included participants over 40 years of age in the CHNS. A total of 4,587 individuals were included in this analysis after excluding missing values and outliers. Simultaneously, we traced the previous screen time to 2004 for those with outcome measures in 2009 (*n* = 2,100) (Figure 1). We included people who did not have a stroke in 2004 and 2006, with the aim of looking at the extent of the risk of stroke in these individuals in the subsequent year 2009.

### 2.2. Anthropometric measurements

Weight, height, waist-to-hip, and blood pressure are measured through a physical examination. Three readings of blood pressure were taken, and the mean of these readings was used. Body mass index (BMI) was calculated by dividing weight (kg) by the square of height (m^2^). A BMI of 28 or higher was considered overweight, while a BMI of 24 or higher and less than 28 was considered obesity (18). Based on the waist-to-hip and waist-to-height ratios, we calculated central adiposity. In our study, central obesity was defined by a waist-to-hip ratio of ≥0.90 in men and ≥0.85 in women (23). A waist-to-height ratio of 0.5 or higher was used to define central obesity in additional analyses (24). All those who carried out the measurements were systematically trained nutritionists with specialized knowledge.

### 2.3. Screen time

The following questions concerning the time of screen were asked: “How much time do you spend on screen behavior from Monday to Friday?” and “How much time do you spend on screen behavior during Saturday to Sunday?” Screen behavior was categorized into (a) watch TV or video; (b) computer use; and (c) gaming. Weekday screen time was calculated as 5 multiplied by the average daily time spent Monday through Friday. Weekend screen time was calculated as two times the average daily time spent on screen from Saturday to Sunday. Total screen time was the sum of weekday and weekend screen time. The daily screen time was the total screen time divided by 7.

### 2.4. Physical activity

The following questions concerning the time of screen were asked: “How much time do you spend on physical activity from Monday to Friday?” and “How much time do you spend on physical activity during Saturday to Sunday?” Physical activity included the following: (a) martial arts; (b) gymnastics, dancing, and acrobatics; (c) track and field, and swimming; (d) walking; (e) soccer, basketball, and tennis; (f) badminton and volleyball; and (g) others (ping pong, etc.). Weekday physical activity was calculated as 5 multiplied by the average daily time spent Monday through Friday. Weekend screen time was calculated as two times the average daily time spent on physical activity from Saturday to Sunday. Total physical activity was the sum of weekday and weekend screen time. Total metabolic equivalent (MET) is the sum of MET of each physical activity above.

### 2.5. Energy intake

CHNS used three consecutive 24-h dietary reviews to measure energy intake. Participants recorded the type and amount of all food and beverages consumed at home and out of home during 24 h on three consecutive days (25). The energy of each food item was calculated based on the China food composition table (26). The daily energy intake for each participant was calculated as the 3-day energy intake divided by 3. Participants who reported a total daily energy intake of more than 5,000 kcal were considered as outliers and were excluded.

### 2.6. Covariates

Self-reported sociodemographic characteristics included gender, age and education level, occupation, etc. According to the criteria of the Implementation Plan of the Pilot Project on Screening and Intervention for High-Risk Stroke Populations in China (27), the participants were classified into low-, middle-, and high-risk groups. Stroke risk factors include the following: (a) history of hypertension; (b) heart disease; (c) smoking; (d) dyslipidemia; (e) diabetes; (f) rarely engage in physical activity (the criteria for physical activity are ≥3 times a week, ≥30 min each time. People engaged in moderate or heavy physical labor are considered to have regular physical exercise); (g) clearly overweight (BMI ≥ 26 kg/m^2^); (h) family history of stroke; (i) history of stroke or transient ischemic attack (TIA). Those with three or more of the first eight stroke risk factors, or those with transient ischemic attacks or a history of previous stroke were included in the high-risk group. Participants with less than three risk factors but with at least hypertension, diabetes, atrial fibrillation, or heart disease were included in the middle-risk group. Those who with less than three risk factors and do not have any of the above three diseases were included in the low-risk group. The covariates considered as potential confounders in the model included age, zone of residence, education level, occupation, drinking, and energy intake.

### 2.7. Statistical analyses

All data analyses were conducted using SAS procedures (SAS, version 9.4, 2022, SAS Institute Inc., Cary, NC, USA). Normality of all continuous variables was examined by using normal probability plots and the Kolmogorov–Smirnov test. Non-normal continuous variables were expressed as median (25th percentile, 75th percentile). Participants were divided into three groups (<2, 2–3, >3 h) according to the duration of the screen to illustrate its relationship with general characteristics. Participants were divided into three groups according to the risk of stroke (low-, middle-, and high-risk groups). Significant differences for non-normally distributed continuous variables were analyzed with the Kruskal–Wallis test, and categorical variables were analyzed with the chi-square test. Multivariable logistic regression was performed for screen time and risk of stroke. Multivariable logistic regression was performed for screen time and risk of stroke. Using screen time in 2004 and 2006 as independent variables, logistic regression analysis was conducted with stroke risk in 2009 as the dependent variable. *p*-values of <0.05 were considered statistically significant.

## 3. Results

Comparison of basic information between the included participants and the total participants above 40 years old is shown in Supplementary Table S1. We gained insight into the differences and commonalities between the two groups by conducting a comparative analysis of the demographic characteristics of the included groups and the total population. In this study, we collected data including age, gender, education level, etc., and conducted detailed statistics and analyses. No significant differences were found between the included participants and the total participants in terms of age distribution, education level, and zone of residence. In the comparative analyses in terms of gender, we found some subtle differences in the gender ratios of the included group and the total population. For example, the proportion of females in the study group was slightly higher than that of the overall population, probably because females showed higher adherence in the study. In contrast, the gender ratio of the overall population was closer to gender balance.

Basic characteristics of the participants in 2009 in this study are presented by the risk of stroke in Supplementary Table S2. In the present analysis, 54.20% of adults were women and the median age of participants was 57.66 years. Compared with women, men are more likely to be at high risk of stroke. Participants with the highest risk of stroke smoke and drink less, but drink more tea (*p *= 0.038). Participants with the highest risk of stroke had a significantly higher percentage of BMI, waist-to-height ratio, blood pressure, and dyslipidemia compared to participants with the low and middle risk of stroke. The total energy and carbohydrate intake increases with the decrease of the risk of stroke. Participants with the highest risk tend to have more screen time and less physical activity (all *p*-values < 0.0001).

Supplementary Table S3 shows the trend of screen time from 2004 to 2009. The proportion of individuals with more than 3 h of screen time per day increased, from 15.14% in 2004 to 22.42% in 2009, and the proportion of individuals with less than 2 h of screen time per day decreased from 42.71% in 2004 to 33.20% in 2009 (*p* < 0.0001).

Basic characteristics of the participants in 2009 in this study are presented by screen time in Table 1. Of all participants, 3,004 (65.49%) participants spent more than 2 h per day on screen time. With the increase of screen time, the proportion of men gradually increases. As expected, participants with the highest screen time were more likely to smoke, drink alcohol, and had higher BMI than participants with the lowest screen time, and they were more likely to be overweight (all *p*-values < 0.05). Participants with the lowest screen time had a significantly lower percentage of dyslipidemia (*p* < 0.05). The intake of carbohydrate is higher in participants with long screen time, and they tend to have less daily physical activity (*p* < 0.0001).

**Table 1:** Basic characteristics of the participants by screen time in this study in 2009^a^ (*n* = 4,587).

	Screen time			*p*-value
	<2 h/day	2–3 h/day	>3 h/day	
*n* (%)	1,583 (34.51)	2,025 (44.15)	979 (21.34)	—
Female (%)	900 (56.85)	1,095 (54.07)	491 (50.15)	<0.0001
Age^b^ (years)	57 (49, 66)	56 (49, 64)	55 (48, 64)	0.0002
Socio-demographics				
Occupation (manual, %)	737 (46.56)	839 (41.43)	266 (27.17)	<0.0001
Occupation (retiree, %)	729 (46.05)	1,033 (51.01)	550 (56.18)	<0.0001
High education level^c^ (%)	626 (39.54)	999 (49.33)	609 (62.21)	<0.0001
Zone of residence (countryside, %)	1,152 (72.77)	1,474 (67.85)	518 (52.91)	<0.0001
Smoking (current smoking, %)	414 (26.15)	541 (26.71)	313 (31.97)	0.003
Alcohol (yes, %)	469 (29.63)	617 (30.47)	351 (35.85)	0.002
At least once a month	421 (26.59)	559 (27.60)	298 (30.44)	0.1
Tea (yes, %)	489 (30.89)	774 (38.22)	472 (48.21)	<0.0001
More than 2–3 times a week (%)	453 (28.62)	717 (35.40)	445 (45.45)	<0.0001
Physical measurement				
Body mass index (kg/m^2^)	23.15 (20.93, 25.39)	23.5 (21.32, 25.87)	23.9 (21.70, 26.28)	<0.0001
Overweight^d^ (%)	481 (30.39)	681 (33.63)	350 (35.75)	0.01
Obesity^e^ (%)	153 (9.67)	215 (10.62)	125 (12.77)	0.047
Hipline (cm)	94.00 (89.00, 99.00)	95.00 (90.00, 100.00)	96.00 (92.00, 101.00)	<0.0001
Waist (cm)	83.00 (76.20, 90.00)	84.00 (77.00, 90.40)	85.00 (79.00, 92.10)	<0.0001
Waist-to-hip ratio	0.88 (0.84, 0.93)	0.88 (0.84, 0.93)	0.89 (0.84, 0.93)	0.3
Waist-to-height ratio	0.52 (0.48, 0.57)	0.52 (0.48, 0.56)	0.53 (0.49,0.57)	0.016
Centripetal obesity^f^	907 (57.30)	1,134 (56.00)	560 (57.20)	0.7
Centripetal obesity^g^	1,025 (64.75)	1,340 (66.17)	674 (68.85)	0.1
Upper arm circumference (cm)	27.00 (24.5, 29.00)	27.10 (25.00, 29.60)	28.00 (25.70, 30.00)	<0.0001
Triceps skinfold thickness (mm)	15.33 (10.00, 20.67)	16 (10.67, 21.67)	16.33 (11.00, 22.67)	0.0004
Blood pressure				
Systolic blood pressure	126.67 (116.67, 140.00)	125.33 (117.33, 139.33)	126.00 (115, 140.00)	0.6
Diastolic blood pressure	80.00 (73.33, 89.33)	80.67 (74.67, 89.83)	80.33 (74.67, 90.00)	0.6
Blood index				
Total cholesterol (mmol/L)	4.83 (4.24, 5.55)	4.96 (4.36, 5.66)	5.08 (4.44, 5.73)	<0.0001
Triglyceride (mmol/L)	1.29 (0.87, 1.93)	1.34 (0.90, 2.01)	1.44 (1.00, 2.19)	<0.0001
High-density lipoprotein (mmol/L)	1.42 (1.19, 1.68)	1.39 (1.17, 1.64)	1.38 (1.15, 1.63)	0.006
Low-density lipoprotein	2.98 (2.34, 3.30)	3.10 (2.53, 3.37)	3.10 (2.56, 3.72)	<0.0001
Blood glucose level (mmol/L)	5.19 (4.8, 5.72)	5.24 (4.80, 5.80)	5.24 (4.80, 5.80)	<0.0001
Diet information				
Energy intake (kcal/day)	2,061.63 (1,634.43, 2,527.10)	2,055.98 (1,662.06, 2,503.00)	2,074.54 (1,666.57, 2,497.55)	0.9
Protein (g/day)	60.22 (47.92, 78.31)	62.64 (49.93, 79.17)	65.07 (51.27, 80.59)	0.002
Fat (g/day)	66.19 (45.51, 93.57)	69.42 (48.64, 94.25)	72.88 (50.69, 99.51)	0.0001
Carbohydrate (g/day)	283.00 (221.14, 362.14)	278.66 (219.02, 354.85)	267.44 (207.07, 329.01)	<0.0001
Disease				
Hypertension^h^ (yes, %)	611 (38.60)	761 (37.58)	399 (40.76)	0.2
Dyslipidemia^i^ (yes, %)	942 (59.51)	1,296 (64.00)	649 (66.29)	0.001
Atrial fibrillation^j^ (yes, %)	16 (1.01)	26 (1.28)	14 (1.43)	0.6
Diabetes^k^ (yes, %)	141 (8.91)	205 (10.12)	99 (10.11)	0.4
Physical activity				
Sleep time (hours/day)	8.00 (7.00, 8.00)	8.00 (7.00, 8.00)	8.00 (7.00, 8.00)	0.2
MET^l^ (each week)	49.00 (0, 192.5)	40.00 (0, 175.00)	20.00 (0, 112.00)	<0.0001

^a^ Values are median (25th percentile, 75th percentile) or frequencies. Screen time was the sum of time spent on TV and computer use. Significant differences for non-normally distributed continuous variables were analyzed with the Kruskal–Wallis test, and categorical variables were analyzed with the chi-square test.

^b^ Age ≥40 years.

^c^ At least 12 years of school education.

^d^ Body mass index ≥24 kg/m^2^ (18).

^e^ Body mass index ≥28 kg/m^2^ (18).

^f^ Waist-to-hip ratio ≥0.85 (in female); waist-to-hip ratio ≥0.90 (in male) (23).

^g^ Waist-to-height ratio ≥0.5 (24).

^h^ Hypertension was defined as having previous history of hypertension or systolic blood pressure ≥140 mmHg and/or diastolic blood pressure ≥90 mmHg (4).

^i^ Diagnosis of dyslipidemia: total cholesterol ≥6.2 mmol/L, triacylglycerol ≥2.3 mmol/L, high-density lipoprotein cholesterol <1.0 mmol/L, low-density lipoprotein cholesterol ≥4.1 mmol/L, and any of the above is considered as dyslipidemia (6).

^j^ Diagnosed with atrial fibrillation by the doctor.

^k^ Diabetes was defined as measured fasting blood glucose ≥7.0 mmol/L or self-reported diagnosis of diabetes (5).

^l^ Total metabolic equivalent is the sum of metabolic equivalent (MET) of each physical activity above.

Association between screen time and the risk of stroke in 2009 is presented in Table 2 and Supplementary Table S4. Compared with the participants who spent less than 2 h on screen per day, the crude odds ratio (OR) of the high risk of stroke was 1.24 [95% confidence interval (CI), 1.05–1.45] for the participants who spent 2–3 h per day on screen and 1.61 (95% CI = 1.33–1.95) for the participants who spent more than 3 h per day on screen. This difference remained significant after adjusting for confounding factors.

**Table 2:** Logistic regression for the association of screen time and the risk of stroke in 2009 (*n* = 4,587).

	Screen time		
	<2 h/day (*n* = 1,583)	2–3 h/day (*n* = 2,025)	>3 h/day (*n* = 979)
Middle risk	*n* = 500	*n* = 632	*n* = 285
Model A	1.00	1.09 (0.93–1.28)	1.14 (0.93–1.40)
Model B	1.00	1.09 (0.92–1.28)	1.15 (0.93–1.43)
Model C	1.00	1.09 (0.92–1.28)	1.15 (0.93–1.42)
Model D	1.00	1.08 (0.92–1.28)	1.11 (0.89–1.37)
High risk	*n* = 509	*n* = 728	*n* = 408
Model A	1.00	1.24 (1.05–1.45)	1.61 (1.33–1.95)
Model B	1.00	1.21 (1,02–1.44)	1.55 (1.25–1.93)
Model C	1.00	1.21 (1.02–1.44)	1.55 (1.24–1.92)
Model D	1.00	1.20 (1.01–1.43)	1.41 (1.13–1.76)

Model A was not adjusted for any variable. Model B was adjusted for age, zone of residence, education level, and occupation. Model C was the same as model B and additionally adjusted for drinking. Model D was the same as model C and additionally adjusted for total energy intake.

Supplementary Tables S5 and S6 show the association between screen time and the risk of stroke by gender in 2009. Compared with the men who spent less than 2 h on screen per day, the crude OR of the middle risk of stroke was 1.29 (95% CI = 1.01–1.63) for the men who spent 2–3 h per day on screen. After adjusting for potential confounders, this association disappeared. Compared with the men who spent less than 2 h on screen per day, the crude OR of the high risk of stroke was 1.53 (95% CI = 1.20–1.95) for the men who spent 2–3 h a day on screen and 2.37 (95% CI = 1.78–3.16) for the men who spent more than 3 h a day on screen. This difference remained significant after adjusting for potential confounders.

Table 3 and Supplementary Tables S7–S9 show the association of screen time in 2006 and the risk of stroke in 2009. The crude OR of the high risk of stroke was 1.36 (95% CI = 1.05–1.78) for the women who spent 2–3 h per day on screen compared with the women who spent less than 2 h on screen per day. After adjusting for potential confounders, this difference remained significant.

**Table 3:** Logistic regression for the association of screen time in 2006 and the risk of stroke in 2009 (*n* = 2,100).

	Screen time		
	<2 h/day (*n* = 824)	2–3 h/day (*n* = 967)	>3 h/day (*n* = 309)
Middle risk	*n* = 112	*n* = 134	*n* = 30
Model A	1.000	1.14 (0.86–1.51)	0.81 (0.52–1.27)
Model B	1.000	1.32 (0.98–1.77)	0.98 (0.61–1.56)
Model C	1.000	1.32 (0.98–1.77)	0.98 (0.61–1.56)
Model D	1.000	1.33 (0.99–1.78)	1.02 (0.64–1.63)
High risk	*n* = 304	*n* = 404	*n* = 144
Model A	1.000	1.26 (1.03–1.55)	1.43 (1.08–1.89)
Model B	1.000	1.26 (1.01–1.57)	1.38 (1.02–1.88)
Model C	1.000	1.26 (1.01–1.57)	1.38 (1.02–1.88)
Model D	1.000	1.26 (1.01–1.57)	1.35 (0.995–1.84)

Model A was not adjusted for any variable. Model B was adjusted for age, zone of residence, education level, and occupation. Model C was the same as model B and additionally adjusted for drinking. Model D was the same as model C and additionally adjusted for total energy intake.

Supplementary Tables S10–S13 show the association between screen time in 2004 and the risk of stroke by gender in 2009. After adjusting for potential confounders, the crude OR of the high risk of stroke was 1.39 (95% CI = 1.04–1.87) for the women who spent 2–3 h per day on screen compared with the women who spent less than 2 h on screen per day. No association was observed between screen time in 2004 and the risk of stroke in men in 2009.

## 4. Discussion

In our study, we evaluated the association between screen time and the risk of stroke. The present study suggested that time spent on screen was positively associated with the risk of stroke in men. In the cohort study, this association was also found in women. This relationship between screen time and stroke remained significant even after adjusting for age, zone of residence, education level, occupation, drinking, and energy intake.

In the present study, the proportion of people at high risk of stroke who are engaged in mental work is 4.6 times higher than those who are engaged in manual work, which may be related to the fact that they tend to have higher levels of sedentary and screen time. The higher energy intake of people at low risk of stroke may also be related to the fact that they have more manual work. The prevalence of hypertension and dyslipidemia among people at high risk of stroke reached 74.7% and 88.3%, respectively. They are all important risk factors for stroke attacks. This study found that people with more than 3 h of screen time tend to have poor lifestyle habits, such as smoking, alcohol consumption, more energy intake, lack of physical activity, and the resulting overweight and obesity problems.

The median amount of screen time observed in this study (2 h/day) was equal to the median amount of TV viewing among older adults in Japan in 2010 (2 h/day) (28), but lower than in Australia (3.5–4 h/day) (12). The prevalence of spending two or more hours a day on screen in this study (65.5%) was consistent with the US results (66.1%) (29), but lower than in the UK (89.2%) (30). In the present study, from 2004 to 2009, participants’ mean screen time increased from 2.0 to 2.5 h/day. From 2004 to 2014, the average amount of time Finns spent watching TV per day gradually increased from 167 to 184 min (31). Therefore, our data may indicate that the amount of time people spend watching TV or using computers is increasing.

Although several articles (21, 32, 33) have focused on the effects of sedentary behavior on stroke, the relationship between screen behavior (17, 34, 35), which accounts for the majority of sedentary time, and stroke remains controversial. Some studies have reported an independent association between TV viewing time and CVD mortality (12, 16). There are many studies that correlate sedentary behavior or screen time with stroke risk factors [hypertension, diabetes (12), hyperlipidemia, heart disease (15), overweight (14), etc.]. Screen behavior may influence stroke by affecting these risk factors. The main reason for the increase in these studies is that sedentary lifestyles have become the new norm in the global workplace (36). In the present analysis, adjustment for age, gender, smoking, the education level, total energy intake, and physical activity enhanced the correlation between screen time and stroke, which confirms that they are important confounders. Among our participants, screen time was related to stroke among men, independent of these confounders.

Multiple logistic regression analysis was performed with the screen time in 2004, 2006, and 2009 as the independent variable and the risk of stroke in 2009 as the outcome, respectively. The difference in the results may be due to the inconsistency in the types of viewing behavior included in the three surveys of viewing time. In 2004, only TV time was included; in 2006, computer time was added; and in 2009, smartphone time was added. Both 2006 and 2009 data showed correlation between screen time and stroke. The association was more pronounced among men. Sedentary behavior is more common among men than women (37). In developing countries, the proportion of obese women is higher than that of men (14). However, in developed countries, more men than women are overweight (38). Some studies have found that men have a higher risk of stroke (35, 39) and dyslipidemia than women (40, 41), and men smoke five times as much as women (42), but more women than men die from CVD in total (43). The reason for this difference may be due to differences in eating habits, lifestyle (44), and hormone levels. Screen behavior tends to be a passive sedentary behavior associated with higher likelihood of snacking, disturbed eating times, and reduced exercise (45).

The present study had several considerable strengths, including the large sample size, high participation rate, repetitive anthropometric measurements, and detailed information on sociodemographic, lifestyle, and dietary data collected by trained workers in face-to-face interviews. Our research population comes from the whole country and includes multiple age groups. The representativeness of this sample for Chinese people in general may have increased the generalizability of the findings. A further strength lies in the adjustment for a number of confounders that potentially affected the associations between screen time or physical activity and the risk of stroke, particularly age, education level, and total energy intake. This study used both cross-sectional analysis and cohort analysis to make the results more convincing.

Some limitations of the present study should be mentioned. There may be measurement errors due to recall bias when evaluating screen use through questionnaires. In addition, due to the annual leave of adults, the screen time in different seasons of the year may also be different. Compared with objective measurement, self-reported screen time usually underestimates the actual screen time. However, as stated in this study, if trained personnel conduct three measurements, the variability within and between observers will be significantly reduced. Although the factors of age, the education level, and total energy intake have been adjusted, there may still be unmeasured or residual confounding.

## 5. Conclusions

We found that the high screen time was associated with an increased stroke risk, which was pronounced in men. Older adults are more susceptible to screen behavior than younger adults due to their fragile health status. We should conduct further studies on stroke in older adults by sex to confirm our results and further explore causality.

## 6. Public health

Our findings have important public health implications as they suggest that screen behavior may play an important role in the development and prevention of stroke, warranting a universal need to limit screen time in order to improve health.

## Data Availability

Publicly available datasets were analyzed in this study. These data can be found here: https://www.cpc.unc.edu/projects/china.
