# Supplementary material for: Association between daily screen time and risk of stroke among middle-aged and elderly people: research based on China health and nutrition survey
Source: Front Sports Act Living. 2023 Dec 13;5:1307930. doi: 10.3389/fspor.2023.1307930 (PMC10751340; doi:10.3389/fspor.2023.1307930)
Supplement: Supplementary file 1 [file Dataheet1.docx]

**Table S1** Comparison of basic information between the included participants and the total participants above 40 years old^1^ (n=13957).

|  | Total participants | Included participants | p value |
| --- | --- | --- | --- |
| n | 13957 | 4587 | - |
| Female (%) | 7256 (52.00) | 2486 (54.20) | 0.009 |
| Age ^2^ (years) | 56.00 (48.00, 65.00) | 56.00 (49.00, 65.00) | 0.82 |
| High education level^3^ (%) | 2741 (19.64) | 934 (20.36) | 0.29 |
| Zone of residence (countryside, %) | 9283 (66.51) | 3044 (66.36) | 0.85 |
| Smoking (current smoking, %) | 3997 (28.63) | 1268 (27.64) | 0.20 |
| Alcohol (yes, %)  At least once a month | 4546 (32.57)  3976 (28.49) | 1437 (31.33)  1278 (27.86) | 0.12  0.41 |

1 Values are median (25th percentile, 75th percentile) or frequencies. Significant differences for non-normally distributed continuous variables were analyzed with the Wilcoxon rank-sum test, and categorical variables were analyzed with the Chi-square test.

2 age ≥45 years.

3 At least 12 years of school education.

**Table S2** Basic characteristics of the participants by the risk of stroke in this study in 2009^1^ (n=4587)

|  | Stroke | | | p value |
| --- | --- | --- | --- | --- |
|  | Low risk | Middle risk | High risk |  |
| n (%) | 1525 (33.25) | 1417 (30.89) | 1645 (35.86) | — |
| Female (%) | 818 (53.60) | 746 (52.60) | 491 (50.15) | <0.0001 |
| Age ^2^ (years) | 53 (46, 60) | 56 (49, 65) | 61 (53, 69) | <0.0001 |
| **Socio-demographics** |  |  |  |  |
| Occupation (manual, %) | 948 (62.16) | 591 (41.71) | 293 (17.81) | <0.0001 |
| High education level^3^ (%) | 319 (20.91) | 302 (21.31) | 313 (19.03) | 0.2 |
| Zone of residence (countryside, %) | 1101 (72.20) | 988 (69.72) | 955 (58.05) | <0.0001 |
| Smoking (current smoking, %) | 491 (32.20) | 417 (29.43) | 360 (21.88) | <0.0001 |
| Alcohol (yes, %)  At least once a month | 519 (34.03)  447 (29.31) | 464 (32.75)  427 (30.13) | 454 (27.60)  404 (24.56) | <0.001  0.001 |
| tea (%)  More than 2-3 times a week (%) | 544 (35.67)  500 (32.79) | 541 (38.18)  505 (35.64) | 650 (39.51)  610 (37.08) | 0.08  0.038 |
| **Physical measurement** |  |  |  |  |
| Body mass index (kg/m^2^)  Overweight ^4^(%)  Obesity ^5^(%) | 21.61 (20.09, 23.05)  158 (10.36)  20 (1.31) | 23.33 (21.22, 25.25)  460 (32.46)  107 (7.55) | 24.09 (25.64, 27.64)  894 (54.35)  366 (22.25) | <0.0001  <0.0001  <0.0001 |
| Hip (cm) | 91.00 (87.00, 95.00) | 95.00 (90.00, 99.00) | 99.00 (94.00, 103.20) | <0.0001 |
| Waist (cm) | 78.70 (73.50, 84.10) | 83.00 (77.00, 90.00) | 90.00 (84.00, 96.00) | <0.0001 |
| Waist to hip ratio | 0.86 (0.82, 0.90) | 0.88 (0.83, 0.92) | 0.91 (0.87, 0.95) | <0.0001 |
| Waist to height ratio | 0.49 (0.46, 0.52) | 0.52 (0.48, 0.56) | 0.56 (0.53, 0.60) | <0.0001 |
| Centripetal obesity^6^ | 845 (55.41) | 967 (68.24) | 1371 (83.34) | <0.0001 |
| Centripetal obesity^7^ | 675 (44.26) | 912 (64.36) | 1443 (87.72) | <0.0001 |
| Upper arm circumference (cm) | 26.00 (24.00, 28.00) | 27.00 (25.00, 29.70) | 28.50 (26.00, 31.00) | <0.0001 |
| Triceps skinfold thickness (mm) | 14.00 (9.00, 19.67) | 15.33 (10.33, 21.00) | 18.00 (13.00, 24.00) | <0.0001 |
| Blood pressure  Systolic blood pressure (mmHg)  Diastolic blood pressure (mmHg) | 118.00 (110, 123.33)  77.33 (70.00, 80.00) | 126.67 (118.67, 140.0)  80.67 (76.00, 90.00) | 140 (126.67, 150.00)  87.33 (80.92, 67.00) | <0.0001  <0.0001 |
| **Blood index** |  |  |  |  |
| Total cholesterol (mmol/l) | 4.64 (4.11, 5.16) | 4.96 (4.35, 5.66) | 5.28 (4.65, 5.94) | <0.0001 |
| Triglyceride (mmol/l) | 1.00 (0.74, 1.40) | 1.34 (0.93, 1.99) | 1.81 (1.23, 2.65) | <0.0001 |
| high-density lipoprotein (mmol/l) | 1.50 (1.28, 1.74) | 1.41 (1.20, 1.66) | 1.27 (1.08, 1.52) | <0.0001 |
| low-density lipoprotein | 2.80 (2.34, 3.30) | 3.06 (2.44, 3.71) | 3.36 (2.70, 3.99) | <0.0001 |
| Blood glucose level (mmol/L) | 5.00 (4.66, 5.40) | 5.14 (4.80, 5.70) | 5.52 (5.00, 6.40) | <0.0001 |
| **Diet information** |  |  |  |  |
| Energy intake (kcal/day) (1 kcal = 4.186kJ) | 2138.70 (1707.22, 2622.83) | 2035.78 (1646.31, 2496.49) | 2009.64 (1623.95, 2423.09) | <0.0001 |
| Protein (g/day) | 62.84 (50.57, 78.87) | 62.22 (49.27, 79.09) | 61.25 (48.83, 79.17) | 0.3 |
| Fat (g/day) | 68.15 (47.56, 98.26) | 68.82 (48.20, 93.07) | 70.61 (48.00, 94.24) | 0.7 |
| carbohydrate (g/day) | 297.88 (235.20, 377.70) | 277.88 (218.60, 348.53) | 260.07 (205.59, 328.44) | <0.0001 |
| **Disease** |  |  |  |  |
| Hypertension ^8^ (yes, %) | 0 | 542 (28.25) | 1229 (74.71) | <0.0001 |
| Dyslipidemia ^9^ (yes, %) | 525 (34.43) | 909 (64.15) | 1453 (88.33) | <0.0001 |
| Atrial fibrillation ^10^ (yes, %) | 0 | 3 (0.21) | 53 (3.22) | <0.0001 |
| Diabetes ^11^ (yes, %) | 0 | 74 (5.22) | 371 (22.55) | <0.0001 |
| **Physical activity** |  |  |  |  |
| Sleep time (hours/day) | 8.00 (7.00, 8.00) | 8.00 (7.00, 8.00) | 8.00 (7.00, 8.00) | 0.2 |
| Screen time ^12^ (hours/day) | 2.00 (1.00, 3.00) | 2.00 (1.10, 3.00) | 2.00 (1.40, 3.00) | <0.0001 |
| Screen time (<2hours/day, %) | 574 (37.64) | 500 (35.29) | 509 (30.94) | <0.0001 |
| Screen time (2~3hours/day, %) | 500 (32.79) | 462 (32.60) | 530 (32.22) |  |
| Screen time (>3hours/day, %) | 451 (29.57) | 455 (32.11) | 606 (36.84) |  |
| Sitting time (hours/day) | 2.00 (1.14, 3.00) | 2.00 (1.33, 3.21) | 2.29 (1.50, 3.57) | <0.0001 |
| Sitting time (<2hours/day, %) | 542 (35.54) | 457 (32.25) | 475 (28.88) | <0.0001 |
| Sitting time (2~3hours/day, %) | 631 (41.38) | 591 (41.71) | 653 (39.70) |  |
| Sitting time (>3hours/day, %) | 352 (23.08) | 369 (26.04) | 517 (31.43) |  |
| MET ^13^ (each week) | 128.00 (35.00, 240.00) | 49.00 (0, 175.13) | 0 (0, 36.00) | <0.0001 |

^1^ Values are median (25th percentile, 75th percentile) or frequencies. Significant differences for non-normally distributed continuous variables were analyzed with the Kruskal-Wallis test, and categorical variables were analyzed with the Chi-square test.

^2^ age≥45.

^3^ At least 12 years of school education.

^4^ Body mass index≥24 kg/m^2^ [18].

^5^ Body mass index≥28 kg/m^2^ [18].

^6^ Waist-hip ratio≥0.85(in female), Waist-hip ratio≥0.90(in male) [23].

^7^ Waist to height ratio≥0.5 [24].

^8^ Hypertension was defined as having previous history of hypertension or systolic blood pressure ≥140 mmHg and/or diastolic blood pressure ≥90 mmHg [4].

^9^ Diagnosis of dyslipidemia: total cholesterol≥6.2 mmol/L, triacylglycerol≥2.3 mmol/L, High-density lipoprotein cholesterol<1.0 mmol/L, Low-density lipoprotein cholesterol≥4.1 mmol/L, and any of the above is considered as dyslipidemia [6].

^10^ Diagnosed with atrial fibrillation by the doctor.

^11^ Diabetes was defined as measured fasting blood glucose ≥7.0 mmol/L or self-reported diagnosis of diabetes [5].

^12^.Screen time were the sum of time spent on TV viewing and computer use.

^13^. Total metabolic equivalent is the sum of metabolic equivalent (MET) of each physical activity above.

**Table S3** The trend of screen time from 2004 to 2009

|  | Screen time | | | p value |
| --- | --- | --- | --- | --- |
|  | <2hous/day | 2-3hours/day | >3hours/day |  |
| 2004, n (%) | 897 (42.71) | 885 (42.14) | 318 (15.14) | <0.0001 |
| 2006, n (%) | 824 (39.23) | 967 (46.05) | 309 (14.71) |  |
| 2009, n (%) | 697 (33.20) | 932 (44.38) | 471 (22.42) |  |

**Table S4** Logistic regression for the association of screen time and the risk of stroke in 2009 (n=4587). (Odds ratios (OR) and 95 % confidence intervals)

|  | Screen time | | |
| --- | --- | --- | --- |
|  | <2hours/day  (n = 1583) | 2-3ours/day  (n = 2025) | >3hours/day  (n = 979) |
| Middle risk | n = 500 | n = 632 | n = 285 |
| Model A | 1.00 | 1.09 (0.93, 1.28) | 1.14 (0.93, 1.40) |
| Model B | 1.00 | 1.09 (0.92, 1.28) | 1.15 (0.93, 1.43) |
| Model C | 1.00 | 1.08 (0.92, 1.28) | 1.11 (0.89, 1.38) |
| Model D | 1.00 | 1.04 (0.87, 1.24) | 1.13 (0.90, 1.42) |
| High risk | n = 509 | n = 728 | n = 408 |
| Model A | 1.00 | 1.24 (1.05, 1.45) | 1.61 (1.33, 1.95) |
| Model B | 1.00 | 1.21 (1,02, 1.44) | 1.55 (1.25, 1.93) |
| Model C | 1.00 | 1.20 (1.00, 1.43) | 1.41 (1.12, 1.76) |
| Model D | 1.00 | 1.09 (0.88, 1.35) | 1.46 (1.12, 1.35) |

Model A was not adjusted for any variable.

Model B was adjusted for age, zone of residence, education level and occupation.

Model C was the same as model B and additionally adjusted for smoking, drinking, total energy intake and physical activity (If MET≤10, they were assigned a value of 10).

Model D was the same as model C and additionally adjusted for blood glucose level and blood lipid level.

**Table S5** Logistic regression for the association of screen time and the risk of stroke by gender in 2009 (n=4587). (Odds ratios (OR) and 95 % confidence intervals)

|  | Screen time | | |
| --- | --- | --- | --- |
|  | <2 hours/day  (n = 1583) | 2-3 hours/day  (n = 2025) | >3 hours/day  (n = 979) |
| Female | n = 900 | n = 1095 | n = 491 |
| Middle risk | n = 280 | n = 321 | n = 145 |
| Model A | 1.00 | 0.95 (0.76, 1.18) | 1.01 (0.76, 1.33) |
| Model B | 1.00 | 0.99 (0.79, 1.25) | 1.00 (0.74, 1.36) |
| Model C | 1.00 | 0.99 (0.79, 1.25) | 1.01 (0.75, 1.37) |
| Model D | 1.00 | 0.99 (0.79, 1.25) | 1.00 (0.74, 1.36) |
| High risk | n = 320 | n = 410 | n = 192 |
| Model A | 1.00 | 1.06 (0.86, 1.31) | 1.17 (0.90, 1.52) |
| Model B | 1.00 | 1.20 (0.94, 1.53) | 1.17 (0.86, 1.60) |
| Model C | 1.00 | 1.20 (0.94, 1.53) | 1.17 (0.86, 1.60) |
| Model D | 1.00 | 1.21 (0.94, 1.54) | 1.12 (0.83, 1.54) |
| Male | n = 683 | n = 930 | n = 488 |
| Middle risk | n = 220 | n = 311 | n = 140 |
| Model A | 1.00 | 1.29 (1.01, 1.63) | 1.32 (0.98, 1.78) |
| Model B | 1.00 | 1.24 (0.97, 1.57) | 1.31 (0.96, 1.78) |
| Model C | 1.00 | 1.24 (0.97, 1.57) | 1.30 (0.96, 1.77) |
| Model D | 1.00 | 1.21 (0.95, 1.54) | 1.20 (0.88, 1.65) |
| High risk | n = 189 | n = 318 | n = 216 |
| Model A | 1.00 | 1.53 (1.20, 1.95) | 2.37 (1.78, 3.16) |
| Model B | 1.00 | 1.31 (1.01, 1.70) | 2.01 (1.47, 2.75) |
| Model C | 1.00 | 1.31 (1.01, 1.69) | 2.01 (1.47, 2.74) |
| Model D | 1.00 | 1.26 (0.97, 1.64) | 1.76 (1.28, 2.41) |

Model A was not adjusted for any variable.

Model B was adjusted for age, zone of residence, education level and occupation.

Model C was the same as model B and additionally adjusted for drinking.

Model D was the same as model C and additionally adjusted for total energy intake.

**Table S6** Logistic regression for the association of screen time and the risk of stroke by gender in 2009 (n=4587). (Odds ratios (OR) and 95 % confidence intervals)

|  | Screen time | | |
| --- | --- | --- | --- |
|  | <2 hours/day  (n = 1583) | 2-3 hours/day  (n = 2025) | >3 hours/day  (n = 979) |
| Female | n = 900 | n = 1095 | n = 491 |
| Middle risk | n = 280 | n = 321 | n = 145 |
| Model A | 1.00 | 0.95 (0.76, 1.18) | 1.01 (0.76, 1.33) |
| Model B | 1.00 | 0.99 (0.79, 1.25) | 1.00 (0.74, 1.36) |
| Model C | 1.00 | 1.00 (0.79, 1.26) | 1.01 (0.74, 1.36) |
| Model D | 1.00 | 0.90 (0.70, 1.15) | 1.01 (0.73, 1.40) |
| High risk | n = 320 | n = 410 | n = 192 |
| Model A | 1.00 | 1.06 (0.86, 1.31) | 1.17 (0.90, 1.52) |
| Model B | 1.00 | 1.20 (0.94, 1.53) | 1.17 (0.86, 1.60) |
| Model C | 1.00 | 1.20 (0.94, 1.54) | 1.12 (0.82, 1.54) |
| Model D | 1.00 | 1.05 (0.78, 1.42) | 1.18 (0.81, 1.74) |
| Male | n = 683 | n = 930 | n = 488 |
| Middle risk | n = 220 | n = 311 | n = 140 |
| Model A | 1.00 | 1.29 (1.01, 1.63) | 1.32 (0.98, 1.78) |
| Model B | 1.00 | 1.24 (0.97, 1.57) | 1.31 (0.96, 1.78) |
| Model C | 1.00 | 1.21 (0.95, 1.54) | 1.21 (0.88, 1.65) |
| Model D | 1.00 | 1.21 (0.94, 1.56) | 1.25 (0.90, 1.74) |
| High risk | n = 189 | n = 318 | n = 216 |
| Model A | 1.00 | 1.53 (1.20, 1.95) | 2.37 (1.78, 3.16) |
| Model B | 1.00 | 1.31 (1.01, 1.70) | 2.01 (1.47, 2.75) |
| Model C | 1.00 | 1.28 (0.97, 1.67) | 1.77 (1.28, 2.45) |
| Model D | 1.00 | 1.16 (0.85, 1.60) | 1.77 (1.21, 2.58) |

Model A was not adjusted for any variable.

Model B was adjusted for age, zone of residence, education level and occupation.

Model C was the same as model B and additionally adjusted for smoking, drinking, total energy intake and physical activity (If MET≤10, they were assigned a value of 10).

Model D was the same as model C and additionally adjusted for blood glucose level and blood lipid level.

**Table S7** Logistic regression for the association of screen time in 2006 and the risk of stroke in 2009 (n=2100). (Odds ratios (OR) and 95 % confidence intervals)

|  | Screen time | | |
| --- | --- | --- | --- |
|  | <2hours/day  (n = 824) | 2-3ours/day  (n = 967) | >3hours/day  (n = 309) |
| Middle risk | n = 112 | n = 134 | n = 30 |
| Model A | 1.000 | 1.14 (0.86, 1.51) | 0.81 (0.52, 1.27) |
| Model B | 1.000 | 1.32 (0.98, 1.77) | 0.98 (0.61, 1.56) |
| Model C | 1.000 | 1.33 (0.99, 1.79) | 1.03 (0.64, 1.64) |
| Model D | 1.000 | 1.31 (0.97, 1.79) | 1.00 (0.61, 1.62) |
| High risk | n = 304 | n = 404 | n = 144 |
| Model A | 1.000 | 1.26 (1.03, 1.55) | 1.43 (1.08, 1.89) |
| Model B | 1.000 | 1.26 (1.01, 1.57) | 1.38 (1.02, 1.88) |
| Model C | 1.000 | 1.26 (1.01, 1.58) | 1.36 (0.99, 1.85) |
| Model D | 1.000 | 1.29 (0.99, 1.66) | 1.44 (1.01, 2.07) |

Model A was not adjusted for any variable.

Model B was adjusted for age, zone of residence, education level and occupation.

Model C was the same as model B and additionally adjusted for smoking, drinking, total energy intake and physical activity (If MET≤10, they were assigned a value of 10).

Model D was the same as model C and additionally adjusted for blood glucose level and blood lipid level.

**Table S8** Logistic regression for the association of screen time in 2006 and the risk of stroke by gender in 2009 (n=2100). (Odds ratios (OR) and 95 % confidence intervals)

|  | Screen time | | |
| --- | --- | --- | --- |
|  | <2hours/day  (n = 824) | 2-3ours/day  (n = 967) | >3hours/day  (n = 309) |
| Female | n = 490 | n = 535 | n = 173 |
| Middle risk | n = 63 | n = 63 | n = 14 |
| Model A | 1.000 | 1.04 (0.70, 1.54) | 0.64 (0.34, 1.21) |
| Model B | 1.000 | 1.22 (0.81, 1.83) | 0.81 (0.42, 1.57) |
| Model C | 1.000 | 1.22 (0.81, 1.83) | 0.81 (0.42, 1.57) |
| Model D | 1.000 | 1.22 (0.81, 1.84) | 0.82 (0.43, 1.60) |
| High risk | n =178 | n = 233 | n = 73 |
| Model A | 1.000 | 1.36 (1.05, 1.78) | 1.19 (0.82, 1.71) |
| Model B | 1.000 | 1.48 (1.10, 1.99) | 1.27 (0.83, 1.93) |
| Model C | 1.000 | 1.48 (1.10, 1.99) | 1.27 (0.83, 1.93) |
| Model D | 1.000 | 1.48 (1.10, 1.99) | 1.27 (0.84, 1.94) |
| Male | n = 334 | n = 432 | n = 136 |
| Middle risk | n = 49 | n = 71 | n = 16 |
| Model A | 1.000 | 1.21 (0.80, 1.85) | 1.06 (0.55, 2.03) |
| Model B | 1.000 | 1.39 (0.90, 2.15) | 1.22 (0.63, 2.38) |
| Model C | 1.000 | 1.40 (0.90, 2.16) | 1.22 (0.62, 2.37) |
| Model D | 1.000 | 1.42 (0.92, 2.19) | 1.30 (0.66, 2.56) |
| High risk | n = 126 | n = 171 | n = 71 |
| Model A | 1.000 | 1.14 (0.83, 1.55) | 1.83 (1.19, 2.82) |
| Model B | 1.000 | 1.05 (0.75, 1.46) | 1.59 (1.01, 2.53) |
| Model C | 1.000 | 1.05 (0.76, 1.47) | 1.59 (0.99, 2.53) |
| Model D | 1.000 | 1.05 (0.75, 1.46) | 1.49 (0.93, 2.37) |

Model A was not adjusted for any variable.

Model B was adjusted for age, zone of residence, education level and occupation.

Model C was the same as model B and additionally adjusted for drinking.

Model D was the same as model C and additionally adjusted for total energy intake.

**Table S9** Logistic regression for the association of screen time in 2006 and the risk of stroke by gender in 2009 (n=2100). (Odds ratios (OR) and 95 % confidence intervals)

|  | Screen time | | |
| --- | --- | --- | --- |
|  | <2hours/day  (n = 824) | 2-3ours/day  (n = 967) | >3hours/day  (n = 309) |
| Female | n = 490 | n = 535 | n = 173 |
| Middle risk | n = 63 | n = 63 | n = 14 |
| Model A | 1.000 | 1.04 (0.70, 1.54) | 0.64 (0.34, 1.21) |
| Model B | 1.000 | 1.22 (0.81, 1.83) | 0.81 (0.42, 1.57) |
| Model C | 1.000 | 1.23 (0.81, 1.85) | 0.82 (0.42, 1.58) |
| Model D | 1.000 | 1.26 (0.82, 1.92) | 0.79 (0.40, 1.55) |
| High risk | n =178 | n = 233 | n = 73 |
| Model A | 1.000 | 1.36 (1.05, 1.78) | 1.19 (0.82, 1.71) |
| Model B | 1.000 | 1.48 (1.10, 1.99) | 1.27 (0.83, 1.93) |
| Model C | 1.000 | 1.51 (1.12, 2.02) | 1.27 (0.83, 1.93) |
| Model D | 1.000 | 1.45 (1.03, 2.05) | 1.25 (0.76, 2.04) |
| Male | n = 334 | n = 432 | n = 136 |
| Middle risk | n = 49 | n = 71 | n = 16 |
| Model A | 1.000 | 1.21 (0.80, 1.85) | 1.06 (0.55, 2.03) |
| Model B | 1.000 | 1.39 (0.90, 2.15) | 1.22 (0.63, 2.38) |
| Model C | 1.000 | 1.42 (0.92, 2.20) | 1.33 (0.68, 2.63) |
| Model D | 1.000 | 1.41 (0.90, 2.22) | 1.39 (0.68, 2.85) |
| High risk | n = 126 | n = 171 | n = 71 |
| Model A | 1.000 | 1.14 (0.83, 1.55) | 1.83 (1.19, 2.82) |
| Model B | 1.000 | 1.05 (0.75, 1.46) | 1.59 (1.01, 2.53) |
| Model C | 1.000 | 1.03 (0.73, 1.46) | 1.50 (0.93, 2.44) |
| Model D | 1.000 | 1.14 (0.76, 1.69) | 1.61 (0.93, 2.79) |

Model A was not adjusted for any variable.

Model B was adjusted for age, zone of residence, education level and occupation.

Model C was the same as model B and additionally adjusted for smoking, drinking, total energy intake and physical activity (If MET≤10, they were assigned a value of 10).

Model D was the same as model C and additionally adjusted for blood glucose level and blood lipid level.

**Table S10** Logistic regression for the association of screen time in 2004 and the risk of stroke in 2009 (n=2100). (Odds ratios (OR) and 95 % confidence intervals)

|  | Screen time | | |
| --- | --- | --- | --- |
|  | <2hours/day  (n = 897) | 2-3ours/day  (n = 885) | >3hours/day  (n = 318) |
| Middle risk | n = 140 | n = 100 | n = 36 |
| Model A | 1.000 | 0.70 (0.52, 0.93) | 0.72 (0.48, 1.09) |
| Model B | 1.000 | 0.77 (0.57, 1.04) | 0.85 (0.56, 1.31) |
| Model C | 1.000 | 0.77 (0.57, 1.04) | 0.85 (0.56, 1.31) |
| Model D | 1.000 | 0.77 (0.57, 1.04) | 0.87 (0.57, 1.33) |
| High risk | n = 349 | n = 367 | n = 136 |
| Model A | 1.000 | 1.03 (0.84, 1.25) | 1.09 (0.83, 1.43) |
| Model B | 1.000 | 1.09 (0.88, 1.36) | 1.09 (0.80, 1.46) |
| Model C | 1.000 | 1.09 (0.88, 1.36) | 1.09 (0.81, 1.47) |
| Model D | 1.000 | 1.08 (0.87, 1.34) | 1.05 (0.77, 1.41) |

Model A was not adjusted for any variable.

Model B was adjusted for age, zone of residence, education level and occupation.

Model C was the same as model B and additionally adjusted for drinking.

Model D was the same as model C and additionally adjusted for total energy intake.

**Table S11** Logistic regression for the association of screen time in 2004 and the risk of stroke in 2009 (n=2100). (Odds ratios (OR) and 95 % confidence intervals)

|  | Screen time | | |
| --- | --- | --- | --- |
|  | <2hours/day  (n = 897) | 2-3ours/day  (n = 885) | >3hours/day  (n = 318) |
| Middle risk | n = 140 | n = 100 | n = 36 |
| Model A | 1.000 | 0.70 (0.52, 0.93) | 0.72 (0.48, 1.09) |
| Model B | 1.000 | 0.77 (0.57, 1.04) | 0.85 (0.56, 1.31) |
| Model C | 1.000 | 0.78 (0.57, 1.05) | 0.87 (0.57, 1.34) |
| Model D | 1.000 | 0.81 (0.60, 1.10) | 0.90 (0.58, 1.40) |
| High risk | n = 349 | n = 367 | n = 136 |
| Model A | 1.000 | 1.03 (0.84, 1.25) | 1.09 (0.83, 1.43) |
| Model B | 1.000 | 1.09 (0.88, 1.36) | 1.09 (0.80, 1.46) |
| Model C | 1.000 | 1.07 (0.86, 1.34) | 1.06 (0.78, 1.43) |
| Model D | 1.000 | 0.90 (0.70, 1.16) | 0.86 (0.61, 1.22) |

Model A was not adjusted for any variable.

Model B was adjusted for age, zone of residence, education level and occupation.

Model C was the same as model B and additionally adjusted for smoking, drinking, total energy intake and physical activity (If MET≤10, they were assigned a value of 10).

Model D was the same as model C and additionally adjusted for blood glucose level and blood lipid level.

**Table S12** Logistic regression for the association of screen time in 2004 and the risk of stroke by gender in 2009 (n=2100). (Odds ratios (OR) and 95 % confidence intervals)

|  | Screen time | | |
| --- | --- | --- | --- |
|  | <2hours/day  (n = 897) | 2-3ours/day  (n = 885) | >3hours/day  (n = 318) |
| Female | n = 533 | n = 491 | n = 174 |
| Middle risk | n = 74 | n = 47 | n = 19 |
| Model A | 1.000 | 0.72 (0.48, 1.07) | 0.83 (0.47, 1.45) |
| Model B | 1.000 | 0.84 (0.55, 1.28) | 1.05 (0.58, 1.89) |
| Model C | 1.000 | 0.84 (0.55, 1.28) | 1.04 (0.58, 1.88) |
| Model D | 1.000 | 0.86 (0.57, 1.32) | 1.08 (0.60, 1.95) |
| High risk | n = 198 | n = 212 | n = 74 |
| Model A | 1.000 | 1.21 (0.93, 1.57) | 1.20 (0.84, 1.74) |
| Model B | 1.000 | 1.41 (1.05, 1.89) | 1.35 (0.90, 2.05) |
| Model C | 1.000 | 1.41 (1.05, 1.88) | 1.35 (0.90, 2.05) |
| Model D | 1.000 | 1.39 (1.04, 1.87) | 1.33 (0.88, 2.02) |
| Male | n = 364 | n = 394 | n = 144 |
| Middle risk | n = 66 | n = 53 | n = 17 |
| Model A | 1.000 | 0.64 (0.42, 0.97) | 0.58 (0.32, 1.07) |
| Model B | 1.000 | 0.66 (0.43, 1.03) | 0.66 (0.36, 1.23) |
| Model C | 1.000 | 0.66 (0.43, 1.02) | 0.67 (0.36, 1.25) |
| Model D | 1.000 | 0.66 (0.43, 1.02) | 0.66 (0.35, 1.24) |
| High risk | n = 151 | n = 155 | n = 62 |
| Model A | 1.000 | 0.81 (0.59, 1.11) | 0.93 (0.61, 1.41) |
| Model B | 1.000 | 0.79 (0.57, 1.09) | 0.84 (0.54, 1.32) |
| Model C | 1.000 | 0.77 (0.56, 1.08) | 0.86 (0.55, 1.35) |
| Model D | 1.000 | 0.77 (0.55, 1.08) | 0.81 (0.52, 1.26) |

Model A was not adjusted for any variable.

Model B was adjusted for age, zone of residence, education level and occupation.

Model C was the same as model B and additionally adjusted for drinking.

Model D was the same as model C and additionally adjusted for total energy intake.

**Table S13** Logistic regression for the association of screen time in 2004 and the risk of stroke by gender in 2009 (n=2100). (Odds ratios (OR) and 95 % confidence intervals)

|  | Screen time | | |
| --- | --- | --- | --- |
|  | <2hours/day  (n = 897) | 2-3ours/day  (n = 885) | >3hours/day  (n = 318) |
| Female | n = 533 | n = 491 | n = 174 |
| Middle risk | n = 74 | n = 47 | n = 19 |
| Model A | 1.000 | 0.72 (0.48, 1.07) | 0.83 (0.47, 1.45) |
| Model B | 1.000 | 0.84 (0.55, 1.28) | 1.05 (0.58, 1.89) |
| Model C | 1.000 | 0.87 (0.57, 1.33) | 1.08 (0.60, 1.96) |
| Model D | 1.000 | 0.89 (0.58, 1.36) | 1.06 (0.58, 1.93) |
| High risk | n = 198 | n = 212 | n = 74 |
| Model A | 1.000 | 1.21 (0.93, 1.57) | 1.20 (0.84, 1.74) |
| Model B | 1.000 | 1.41 (1.05, 1.89) | 1.35 (0.90, 2.05) |
| Model C | 1.000 | 1.39 (1.04, 1.87) | 1.33 (0.88, 2.01) |
| Model D | 1.000 | 1.30 (0.90, 1.87) | 1.15 (0.69, 1.90) |
| Male | n = 364 | n = 394 | n = 144 |
| Middle risk | n = 66 | n = 53 | n = 17 |
| Model A | 1.000 | 0.64 (0.42, 0.97) | 0.58 (0.32, 1.07) |
| Model B | 1.000 | 0.66 (0.43, 1.03) | 0.66 (0.36, 1.23) |
| Model C | 1.000 | 0.66 (0.43, 1.02) | 0.66 (0.35, 1.24) |
| Model D | 1.000 | 0.77 (0.49, 1.20) | 0.64 (0.33, 1.23) |
| High risk | n = 151 | n = 155 | n = 62 |
| Model A | 1.000 | 0.81 (0.59, 1.11) | 0.93 (0.61, 1.41) |
| Model B | 1.000 | 0.79 (0.57, 1.09) | 0.84 (0.54, 1.32) |
| Model C | 1.000 | 0.76 (0.54, 1.07) | 0.82 (0.52, 1.29) |
| Model D | 1.000 | 0.76 (0.51, 1.13) | 0.69 (0.41, 1.17) |

Model A was not adjusted for any variable.

Model B was adjusted for age, zone of residence, education level and occupation.

Model C was the same as model B and additionally adjusted for smoking, drinking, total energy intake and physical activity (If MET≤10, they were assigned a value of 10).

Model D was the same as model C and additionally adjusted for blood glucose level and blood lipid level.
